# Weight Loss Improves Predicted 10‐Year MACE and Type 2 Diabetes Risk, but Benefits Partially Rebound at 1 Year

**DOI:** 10.1002/oby.70291

**Published:** 2026-08-23

**Authors:** Abdelrahman M. Elshakankiry, Gary R. Hunter, Cátia Martins, Gordon Fisher, W. Timothy Garvey, Carrie R. Howell

**Affiliations:** ^1^ Department of Nutrition Sciences, School of Health Professions University of Alabama at Birmingham Birmingham Alabama USA; ^2^ Department of Human Studies University of Alabama at Birmingham Birmingham Alabama USA; ^3^ Department of Medicine, Division of Internal General Medicine and Population Science University of Alabama at Birmingham Birmingham Alabama USA

**Keywords:** cardiometabolic risk, resistance exercise, type 2 diabetes, weight loss, weight regain

## Abstract

**Objective:**

This study aimed to quantify changes in predicted 10‐year risk for major adverse cardiovascular events (MACE) and type 2 diabetes (T2D) across weight loss, post–weight loss (Post‐WL), and 1‐year follow‐up and to determine whether trajectories varied by exercise modality.

**Methods:**

Sedentary women with overweight (21–49 years) were randomized to Diet+Aerobic, Diet+Resistance, or Diet‐only and completed an 800 kcal/day diet until BMI < 25 kg m^−2^. Predicted 10‐year MACE and T2D risks were calculated using cardiometabolic disease staging equations. Longitudinal changes and adherence effects were analyzed using fractional logit models fit with generalized estimating equations.

**Results:**

Among 221 women (115 African American), mean BMI decreased from 28.3 to 23.9 kg m^−2^, with regain to 25.8 at 1 year. MACE risk decreased from Baseline to Post‐WL and rebounded from Post‐WL to 1 year in all groups (all *p <* 0.001). Diet‐only achieved greater MACE reduction than Diet+Resistance (*p =* 0.022). T2D risk decreased in all groups (all *p <* 0.001) but rebounded in Diet+Aerobic and Diet‐only. The Diet+Resistance group maintained a significant net reduction at 1 year (*p =* 0.003). Follow‐up exercise adherence was associated with lower T2D risk rebound (*p =* 0.024).

**Conclusions:**

Weight loss reduced MACE and T2D risks, but both rebounded with weight regain. Resistance exercise may be beneficial in weight loss interventions to prevent progression to T2D.

**Trial Registration:**

ClinicalTrials.gov identifier: NCT00067873

## Study Importance

- What is already known?
  ○ Intentional weight loss improves cardiometabolic health, but partial weight regain is common and may offset these benefits.
  ○ Aerobic and resistance exercise improves metabolic health through distinct mechanisms, but whether these modalities differentially influence composite cardiometabolic risk during weight loss and subsequent weight regain has not been directly quantified.
- What does this study add?
  ○ Using validated cardiometabolic disease staging equations, this study directly quantifies predicted 10‐year major adverse cardiovascular events and type 2 diabetes (T2D) risk trajectories during weight loss, at post–weight loss, and at 1‐year follow‐up in a randomized trial directly comparing Diet+Aerobic, Diet+Resistance, and Diet‐only interventions in premenopausal women with overweight.
  ○ Diet+Resistance was the only arm to maintain a significant net reduction in T2D risk at 1 year despite partial weight regain, and higher follow‐up exercise adherence independently attenuated T2D risk rebound.
- How might these results change the direction of research or the focus of clinical practice?
  ○ Resistance exercise may be beneficial in weight loss interventions aiming to prevent progression to T2D.
  ○ Sustaining exercise following weight reduction is critical for preventing T2D risk rebound, even when some weight regain occurs.

## 1 Introduction

Excessive weight gain in adults is associated with metabolic changes that reflect sustained positive energy balance and worsening cardiometabolic disease risk. These changes are characterized by a reduction in insulin sensitivity, higher fasting and postprandial glucose, an adverse lipid profile with hypertriglyceridemia and low HDL‐C, and increases in blood pressure (BP). These traits cluster as the metabolic syndrome and increase the risk for type 2 diabetes (T2D) as well as cardiovascular disease (CVD) [1]. Importantly, these factors associated with excess adiposity are modifiable and respond to lifestyle interventions, making intentional weight loss and structured exercise crucial for cardiometabolic risk reduction [2, 3].

Weight loss produces broad improvements in metabolic health, largely due to reductions in adiposity and ectopic lipid deposition, in addition to improvements in insulin sensitivity and cardiometabolic risk factor profiles [4]. In individuals at high risk of T2D, lifestyle interventions producing weight loss markedly reduce incident T2D, linking weight reduction and behavioral change to diabetes prevention [5]. Longer‐term follow‐up of these lifestyle programs suggests that benefits may be sustained beyond the initial intervention period, even as weight trajectories vary over time [6]. In patients with diabetes, the Look AHEAD trial also demonstrated that the magnitude and maintenance of weight loss produced by an intensive lifestyle intervention are important determinants of longer‐term cardiometabolic profiles [7, 8]. However, maintaining weight loss is challenging, and weight regain is common. Previous studies have shown that cardiometabolic improvements are attenuated when weight is regained, reinforcing the need to understand the rebound in metabolic risk associated with weight regain following successful weight loss interventions [9, 10].

Exercise complements dietary weight loss by improving metabolic health through mechanisms that are partly independent of changes in adiposity [11, 12]. Aerobic training enhances cardiorespiratory fitness and has favorable effects on several cardiometabolic risk factors such as BP and lipid profile, although the dose, intensity, and duration of exercise influence the magnitude of response [13, 14]. However, the combination of aerobic exercise with modest weight loss appears to yield larger benefits than exercise without weight loss, suggesting additive benefits from combining interventions [15]. Resistance training works differently by increasing or preserving skeletal muscle mass and strength, which may enhance the glucose disposal capacity and metabolic resilience during and after weight loss [16]. Previous work also demonstrated that combined aerobic and resistance training improves glycemic control more than either modality alone and may provide broader cardiometabolic benefits across multiple risk factors [17, 18, 19, 20]. In addition, while aerobic, resistance, and combined training all improve risk factors, the degree of improvement can vary among different CVD risk factor domains, highlighting why comparisons of diet and exercise modalities within a common study design remain valuable [21].

While these studies address the benefits of diet and exercise over the period of intervention, the maintenance of benefits following cessation of the intervention, which is often associated with weight regain, is less well understood. The practical challenge is how to understand the effects of lifestyle interventions when multiple cardiometabolic biomarkers change simultaneously to varying degrees. These combined effects can be better understood when integrated into a single quantitative future‐risk metric. Predicted‐risk models integrate measures such as BP, lipids, and glycemic markers into a single probability metric, thereby capturing the combined contribution of several physiologic markers that change with weight loss and exercise [1, 22]. This approach is particularly helpful for interventions that produce noticeable benefits during active weight loss followed by variable maintenance. Furthermore, the level of adherence with the prescribed exercise during the follow‐up is often heterogeneous, which can explain the individual variations in long‐term metabolic outcomes after weight loss [23].

Therefore, the aim of this secondary analysis was to quantify and compare changes in predicted 10‐year major adverse cardiovascular events (MACE) and T2D risks over time among premenopausal women with overweight randomized to Diet+Aerobic, Diet+Resistance, or Diet‐only and to determine whether aerobic versus resistance training differentially influences risk reduction and rebound. For this purpose, we employed cardiometabolic disease staging (CMDS), which incorporates multiple cardiometabolic risk factors as continuous variables and provides a quantitative measure of the severity of cardiometabolic disease as reflected in robust 10‐year risk estimates for T2D and MACE. We hypothesized that weight loss would reduce predicted 10‐year MACE and T2D risk across all intervention arms. We also hypothesized that partial rebound would occur during the follow‐up period, and that adding structured exercise would be associated with more favorable predicted‐risk trajectories.

## 2 Methods

### 2.1 Participants

Premenopausal women, both African American (AA) and European American (EA), with overweight (27 < BMI < 30 kg m^−2^) were recruited for a study aiming to investigate metabolic factors related to weight gain. Women also needed to be nonsmokers, be in good health, have normal menstrual cycles, be sedentary (≤ 1 structured exercise session/week), and have a normal glucose tolerance measured with a 2‐h oral glucose tolerance test (OGTT). Women were excluded if they were using oral contraceptives at enrollment or taking medications known to affect body composition. All participants provided written informed consent. The protocol was approved by the University of Alabama at Birmingham Institutional Review Board and conducted in accordance with the US Department of Health and Human Services regulations for the protection of human research participants.

### 2.2 Study Design, Time Points, and Outcome Measures

Participants were randomly assigned to one of three weight loss interventions: Diet+Aerobic, Diet+Resistance, or Diet‐only. All testing was conducted under controlled conditions during an inpatient stay at the General Clinical Research Center (GCRC). During weight loss, all participants followed the same 800 kcal/day diet (~58%–62% carbohydrate, 18%–22% protein, and 20%–22% fat) provided by the metabolic kitchen of the GCRC until achieving BMI < 25 kg m^−2^. Assessments occurred at Baseline (overweight state), at post–weight loss (Post‐WL; after achieving BMI < 25 kg m^−2^), and at 1‐year follow‐up. All assessments were done during the follicular phase of the menstrual cycle, following an overnight fast of ≥ 12 h. Participants in the exercise arms underwent inpatient testing ≥ 48 h after the last exercise session. During the year following weight reduction, participants were advised to adhere to a balanced low–energy‐density diet following a set of standardized recommendations based on the EatRight Weight Management Program principles [24]. Additional details on follow‐up dietary guidance, pre‐visit weight stabilization, and inpatient testing procedures at each time point have been published previously [25, 26, 27].

### 2.3 Exercise Intervention

Fifty‐minute training sessions were performed in a research‐dedicated facility at the University of Alabama at Birmingham under the supervision of study personnel. Subjects were required to train three times per week during the weight loss phase and two times per week during the 1‐year follow‐up period. Exercise participation during weight loss was required for continued study enrollment, whereas during follow‐up it was encouraged but not mandatory, resulting in substantial variation in adherence.

### 2.4 Aerobic Training

Aerobic exercise modalities included treadmill walking and running, cycle ergometry, and stair stepping. The first week of training entailed participants completing 20 min of continuous aerobic activity at ~67% HRmax. With each week, the duration and intensity of exercise increased until the end of week 8 when participants were exercising for 40 min at ~80% HRmax, which was maintained thereafter. Further details on the aerobic training progression and target intensity protocol have been published previously [25, 26, 27].

### 2.5 Resistance Training

Participants completed a standardized whole‐body exercise routine that included squats, leg extensions, leg curls, elbow flexions, triceps extensions, lateral pull‐downs, bench presses, military presses, lower back extensions, and bent‐knee sit‐ups. The intensity of the strength training was controlled at 80% of the 1‐RM of each participant, which was increased progressively. Additional details on the resistance training exercises, progression, and intensity have been described previously [25, 26, 27].

### 2.6 Body Weight and Composition

Body composition was measured by dual‐energy X‐ray absorptiometry using a Lunar Prodigy densitometer (GE Medical Systems Lunar, Madison, WI).

### 2.7 Predicted 10‐Year MACE and T2D Risk Calculation

For each measurement time point (Baseline, Post‐WL, and 1‐year follow‐up), participant‐specific predicted 10‐year MACE risk was computed using the validated cardiometabolic disease staging (CMDS) score [28]. The CMDS equations predicting MACE include age, BMI, systolic and diastolic BP, blood glucose, HDL‐C, triglycerides, non‐HDL‐C (total cholesterol minus HDL‐C), diabetes (yes/no), smoking status, and antihypertensive medication use. CMDS scores for MACE were calculated using published formulas and transformed into a probability scale by applying the inverse logit function, yielding a predicted‐risk value bounded between 0 and 1 for each participant at each time point. Predicted 10‐year T2D risk was calculated at the same time points using the corresponding CMDS algorithms incorporating age, sex, race, BMI, systolic and diastolic BP, blood glucose, HDL‐C, and triglycerides [29, 30]. All predictors were entered in the specified units of measure (e.g., glucose, HDL‐C, and triglycerides in mg/dL; BP in mmHg; BMI in kg m^−2^).

### 2.8 Statistical Analysis

Predicted 10‐year MACE and T2D risks were analyzed as fractional outcomes bounded between 0 and 1 using fractional logit models fit with generalized estimating equations (GEE) to account for within‐participant correlation across repeated measures (Baseline, Post‐WL, and 1‐year follow‐up) [31, 32, 33]. Models were fit using a binomial family with logit link, an exchangeable working correlation structure, and robust standard errors (SE) clustered at the participant level. Because follow‐up was incomplete in some participants, models were fit with all available observations, which means that participants could contribute one to three observations across time points, and we did not restrict analyses to complete cases. Therefore, we were able to estimate population‐average changes over time. Valid inference under this approach assumes missing follow‐up is missing at random (MAR). To assess whether attrition was plausibly related to baseline risk, we grouped participants by the number of observed predicted‐risk time points and compared baseline predicted risk across completeness patterns.

Primary longitudinal GEE models included fixed effects for time, intervention group, and a time‐by‐group interaction to estimate group‐specific trajectories. We also tested whether risk reduction (Baseline → Post‐WL), rebound (Post‐WL → 1‐year follow‐up), and net change (Baseline → 1‐year follow‐up) varied by intervention group. Analogous models replacing groups with race evaluated race effects. Model‐estimated risks at each time point (with 95% CI) were obtained from post‐estimation marginal means, and within‐group/race changes were expressed as percentage point differences for risk reduction, rebound, and net change. Between‐group and between‐race differences in change were assessed using linear contrasts of interaction terms, with Bonferroni adjustment applied when families of pairwise comparisons were tested.

To examine whether adherence explained or modified risk changes, time‐window–specific adherence‐adjusted analyses were conducted. Weight loss exercise adherence was evaluated in models restricted to Baseline and Post‐WL, whereas follow‐up exercise adherence and follow‐up diet adherence were evaluated in models restricted to Post‐WL and 1‐year follow‐up. In these models, adherence was included as a continuous covariate scaled per 10% increase, along with time, group, and time‐by‐group. Adherence‐by‐group interactions were also tested to evaluate effect modification by intervention arm. All analyses were performed in Stata version 14 (StataCorp LLC, College Station, TX). Tests were two‐sided and statistical significance was defined as *p <* 0.05.

## 3 Results

Among the 221 women enrolled (115 AA; 106 EA), 212 had sufficient baseline data to estimate predicted risk and were included in the baseline‐risk analytic sample. Across follow‐up, 131 women contributed risk estimates at Post‐WL, and 105 women contributed risk estimates at 1 year. By intervention arm, sample sizes at Baseline, Post‐WL, and 1 year were respectively as follows: Diet+Aerobic (*n* = 82, 44, 31), Diet+Resistance (*n* = 84, 57, 47), and Diet‐only (*n* = 46, 30, 27). A CONSORT flow diagram is provided in Figure S1. Baseline MACE and T2D risk scores were not different as a function of completeness patterns and did not predict dropout prior to Post‐WL or to 1‐year follow‐up (all *p >* 0.05; McFadden pseudo‐*R* ^2^≈0; Table S1). Participant data completeness patterns across time points, including entrants (*n* = 9) who had follow‐up risk estimates but no baseline‐risk data, are summarized in Figure S2. Mean BMI across groups decreased from 28.3 kg m^−2^ at Baseline to 23.9 kg m^−2^ at Post‐WL, with partial regain to 25.8 kg m^−2^ at 1‐year follow‐up. Time required to reach the target BMI averaged 159 ± 72 days and did not differ significantly by group. Anthropometric variables, body composition, and intervention adherence by group and time point are shown in Table 1. Quantitative 10‐year risks are reported as percentages (%), and changes over time are expressed as percentage point (pp) differences.

**TABLE 1:** Anthropometrics, body composition, and adherence over time in the three intervention groups.

	Diet+Aerobic			Diet+Resistance			Diet‐only		
	Baseline (*n* = 82)	Post‐WL (*n* = 44)	1 year (*n* = 31)	Baseline (*n* = 84)	Post‐WL (*n* = 57)	1 year (*n* = 47)	Baseline (*n* = 46)	Post‐WL (*n* = 30)	1 year (*n* = 27)
Age (years)	34.8 ± 6.5	35.4 ± 7.0	35.6 ± 7.1	33.1 ± 5.8	34.4 ± 6.1	35.4 ± 6.3	35.3 ± 5.7	35.4 ± 5.9	37.0 ± 5.6
Race (EA/AA)	41/41	20/24	14/17	41/43	27/30	21/26	23/23	16/14	13/14
BMI (kg m^−2^)	28.6 ± 1.3	23.9 ± 1.1	25.6 ± 2.5	27.9 ± 1.2	23.9 ± 1.0	25.9 ± 1.9	28.5 ± 1.5	23.9 ± 1.1	26.0 ± 1.6
Days to BMI < 25 kg m^−2^	167.7 ± 81.0 (*n* = 44)			158.8 ± 74.8 (*n* = 57)			148.5 ± 50.9 (*n* = 30)		
Weight (kg)	77.0 ± 6.7	64.5 ± 6.2	67.7 ± 8.0	76.8 ± 7.5	66.4 ± 6.6	71.4 ± 7.7	78.0 ± 7.3	66.2 ± 6.2	71.8 ± 7.3
% Body fat	44.3 ± 3.8	33.9 ± 4.7	38.6 ± 5.9	42.5 ± 3.8	32.6 ± 4.7	37.8 ± 5.2	43.1 ± 3.7	33.7 ± 4.7	38.6 ± 5.2
Fat mass (kg)	35.5 ± 4.8	23.1 ± 4.9	26.4 ± 6.5	34.0 ± 5.3	22.7 ± 4.7	27.1 ± 5.5	35.0 ± 5.0	23.4 ± 4.6	27.9 ± 5.9
Fat‐free mass (kg)	42.8 ± 4.3	42.5 ± 3.6	41.2 ± 3.6	44.1 ± 4.2	44.6 ± 4.1	44.3 ± 4.7	44.3 ± 4.2	43.8 ± 4.1	43.9 ± 4.0
Waist circumference (cm)	87.5 ± 6.2	76.1 ± 5.1	80.0 ± 7.6	85.0 ± 6.1	76.1 ± 4.9	79.7 ± 5.9	89.1 ± 7.2	77.2 ± 5.6	81.2 ± 6.8
WL exercise adherence (%)	80.6 ± 17.3 (*n* = 44)			77.3 ± 15.8 (*n* = 57)			—		
Follow‐up exercise adherence (%)	54.7 ± 32.0 (*n* = 31)			56.7 ± 27.0 (*n* = 47)			—		
Follow‐up diet adherence (%)	58.8 ± 30.0 (*n* = 31)			50.4 ± 32.4 (*n* = 45)			59.1 ± 33.3 (*n* = 25)		

*Note:* Values are mean ± SD. Post‐WL: post–weight loss (assessment conducted after achieving BMI < 25 kg m^−2^); 1‐year follow‐up: 1 year after Post‐WL. Race is shown as counts (EA/AA), Exercise adherence is not applicable for Diet‐only (—). Sample sizes for adherence measures may differ from visit *n* due to incomplete adherence data.

Abbreviations: AA, African American; EA, European American.

The 10‐year MACE risk scores decreased significantly from Baseline to Post‐WL in all groups (Diet+Aerobic: −0.14 pp; Diet+Resistance: −0.1 pp; Diet‐only: −0.2 pp; all *p <* 0.001). From Post‐WL to 1‐year follow‐up over the period of weight regain, MACE risk rebounded significantly in all groups (Diet+Aerobic: +0.15 pp; Diet+Resistance: +0.13 pp; Diet‐only: +0.16 pp; all *p <* 0.001). The risk scores for MACE comparing Baseline to 1‐year follow‐up were statistically similar. However, the magnitude of MACE risk reduction after weight loss differed among intervention groups. The Diet‐only arm achieved a greater Baseline‐to–Post‐WL reduction than Diet+Resistance (difference = −0.1 pp; adjusted *p =* 0.022). Other between‐group differences in reduction, rebound, and net change were not statistically significant after multiplicity adjustment. Data from all interventions were combined to assess effects of race, and both EA and AA were found to have significant MACE risk reduction from Baseline to Post‐WL and significant rebound from Post‐WL to 1‐year follow‐up (all *p <* 0.001). Across the full Baseline‐to–1‐year follow‐up interval, net change was not significant for either race (EA: −0.01 pp, *p =* 0.695; AA: +0.03 pp, *p =* 0.271), and between‐race differences in change were not statistically significant for reduction, rebound, or net change.

T2D risk decreased significantly from Baseline to Post‐WL in all groups (Diet+Aerobic: −5.2 pp; Diet+Resistance: −3.6 pp; Diet‐only: −5.2 pp; all *p <* 0.001). During follow‐up, Diet‐only showed significant rebound from Post‐WL to 1‐year follow‐up (+4.0 pp, *p <* 0.001), whereas the risk scores at 1 year did not differ significantly from values at Post‐WL in the Diet+Aerobic (+3.5 pp, *p =* 0.119) and Diet+Resistance (+1.1 pp, *p =* 0.216) arms. However, only the Diet+Resistance group was found to maintain a significant decrease in T2D risk compared to Baseline values at 1‐year follow‐up (Diet+Resistance: −2.4 pp, *p =* 0.003; Diet+Aerobic: −1.6 pp, *p =* 0.436; Diet‐only: −1.2 pp, *p =* 0.311). Between‐group differences in T2D risk change were not statistically significant after multiplicity adjustment.

**FIGURE 1:**
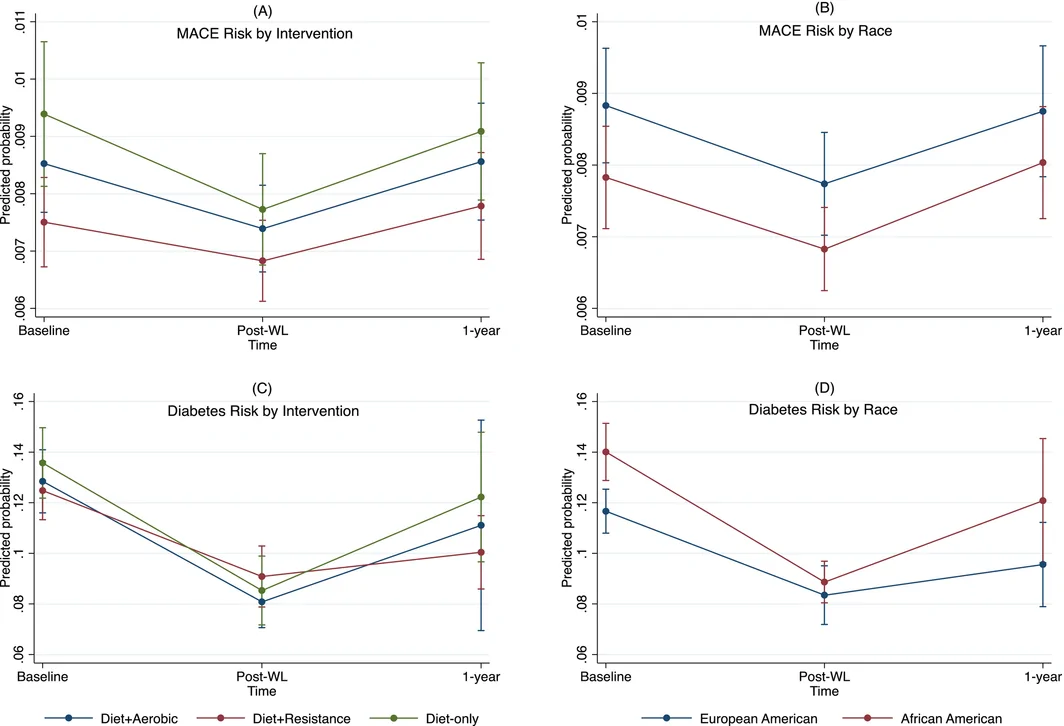
Predicted 10‐year MACE and T2D risk trajectories across time points by intervention group and race. MACE risk by (A) intervention group and (B) race; T2D risk by (C) intervention group and (D) race. Race‐stratified analyses (African American vs. European American) were conducted with intervention arms combined. [Color figure can be viewed at wileyonlinelibrary.com]

When data from all weight loss interventions were combined, both EA and AA showed significant Baseline‐to–Post‐WL reductions in T2D risk (both *p <* 0.001). During follow‐up, AA showed a significant rebound from Post‐WL to 1 year (+3.5 pp, *p =* 0.006), while the increase in risk in EA was less pronounced (+1.5 pp, *p =* 0.152). Furthermore, when 1‐year follow‐up values were compared with Baseline, EA retained a significant net reduction in T2D risk (−2.2 pp, *p =* 0.017), whereas AA did not (−1.7 pp, *p =* 0.153). Between‐race differences in change were not statistically significant for reduction, rebound, or net change. Within‐group and within‐race trajectories for both outcomes are shown in Table 2, whereas between‐group and between‐race contrasts in change over time are presented in Table 3. Predicted 10‐year MACE and T2D risk trajectories by intervention group and race are shown in Figure 1.

**TABLE 2:** Predicted 10‐year MACE and T2D risk over time within groups and races.

	Baseline, % (95% CI)	Post‐WL, % (95% CI)	1 year, %(95% CI)	Δ1 (pp)	*p*	Δ2 (pp)	*p*	Δ3 (pp)	*p*
MACE									
Group effect									
D + A	0.85 (0.77–0.94)	0.74 (0.66–0.82)	0.87 (0.75–0.96)	−0.14	< 0.001	+0.15	< 0.001	+0.00	0.864
D + R	0.75 (0.67–0.83)	0.68 (0.61–0.75)	0.78 (0.69–0.87)	−0.10	< 0.001	+0.13	< 0.001	+0.04	0.181
D	0.94 (0.81–1.1)	0.77 (0.68–0.87)	0.91 (0.79–1.0)	−0.20	< 0.001	+0.16	< 0.001	−0.03	0.331
Race effect									
EA	0.88 (0.80–0.96)	0.77 (0.70–0.85)	0.88 (0.78–0.97)	−0.13	< 0.001	+0.12	< 0.001	−0.00	0.695
AA	0.78 (0.71–0.85)	0.68 (0.63–0.74)	0.80 (0.73–0.88)	−0.14	< 0.001	+0.16	< 0.001	+0.03	0.271
T2D									
Group effect									
D + A	12.9 (11.6–14.1)	8.1 (7.1–9.1)	11.1 (6.7–15.3)	−5.2	< 0.001	+3.5	0.119	−1.6	0.436
D + R	12.5 (11.3–13.6)	9.1 (7.9–10.3)	10.0 (8.6–11.5)	−3.6	< 0.001	+1.1	0.216	−2.4	0.003
D	13.6 (12.2–15.0)	8.5 (7.2–9.9)	12.2 (9.7–14.8)	−5.2	< 0.001	+4.0	< 0.001	−1.2	0.311
Race effect									
EA	11.7 (10.8–12.5)	8.4 (7.2–9.5)	9.6 (7.9–11.2)	−3.7	< 0.001	+1.5	0.152	−2.2	0.017
AA	14.0 (12.9–15.1)	8.9 (8.0–9.7)	12.1 (9.6–14.5)	−5.2	< 0.001	+3.5	0.006	−1.7	0.153

*Note:* Values are model‐estimated predicted probabilities from fractional logit models fit with GEE. Groups are shown as D + A: Diet+Aerobic, D + R: Diet+Resistance, D: Diet‐only. Differences are percentage point (pp) changes. Δ1: Post‐WL–Baseline (pp); Δ2: 1‐year follow‐up−Post‐WL (pp); Δ3: 1‐year follow‐up−Baseline (pp).

Abbreviations: AA, African American; EA, European American; WL, weight loss.

**TABLE 3:** Predicted 10‐year MACE and T2D risk over time ‐ Between‐group and race differences in change.

Contrast (Δ change)	Estimate (pp)	SE (pp)	*z*	*p* raw (*p* adjusted)
MACE				
Risk reduction (Baseline → Post‐WL)				
Diet+Resistance – Diet+Aerobic	+0.05	0.02	1.96	0.051 (0.304)
Diet‐only – Diet+Aerobic	−0.05	0.04	−1.43	0.153
Diet‐only – Diet+Resistance	−0.10	0.03	−2.90	0.004 (0.022*)
AA – EA	−0.44	2.9	−0.15	0.878
Risk rebound (Post‐WL → 1 year)				
Diet+Resistance – Diet+Aerobic	−0.02	0.03	−0.60	0.550
Diet‐only – Diet+Aerobic	+0.02	0.04	+0.50	0.619
Diet‐only – Diet+Resistance	+0.04	0.03	+1.22	0.222
AA – EA	+4.0	3.3	+1.20	0.228
Net change (Baseline → 1 year)				
Diet+Resistance – Diet+Aerobic	+0.03	0.03	+0.83	0.405
Diet‐only – Diet+Aerobic	−0.03	0.04	−0.90	0.366
Diet‐only – Diet+Resistance	−0.06	0.04	−1.54	0.123
AA – EA	+3.6	3.4	+1.06	0.288

T2D				
Risk reduction (Baseline → Post‐WL)				
Diet+Resistance – Diet+Aerobic	+1.4	0.83	1.64	0.100
Diet‐only – Diet+Aerobic	−0.28	0.98	−0.28	0.776
Diet‐only – Diet+Resistance	−1.6	1.0	−1.61	0.108
AA – EA	−14.4	8.4	−1.72	0.085
Risk rebound (Post‐WL → 1 year)				
Diet+Resistance – Diet+Aerobic	−2.1	2.3	−0.89	0.372
Diet‐only – Diet+Aerobic	+0.67	2.5	0.27	0.787
Diet‐only – Diet+Resistance	+2.7	1.4	1.94	0.052 (0.313)
AA – EA	+19.7	16.3	1.21	0.226
Net change (Baseline → 1 year)				
Diet+Resistance – Diet+Aerobic	−0.70	2.2	−0.31	0.754
Diet‐only – Diet+Aerobic	+0.39	2.5	0.16	0.874
Diet‐only – Diet+Resistance	+1.1	1.5	0.73	0.467
AA – EA	+5.3	15.1	0.35	0.728

*Note: p* values are presented as raw and (Bonferroni‐adjusted) for multiple comparisons within each family of tests; *indicates *p <* 0.05 after adjustment.

Abbreviations: AA, African American; EA, European American; WL, weight loss.

Adherence to the prescribed interventions was higher during the weight loss phase than during follow‐up. Exercise adherence, reflecting attendance at the prescribed sessions in the Diet+Aerobic and Diet+Resistance arms, averaged 78.7% ± 16.5% during weight loss (*n* = 101). During follow‐up, exercise adherence averaged 55.9% ± 28.9% (*n* = 78), and diet adherence averaged 55.1% ± 31.9% (*n* = 101). In time‐window–specific fractional logit GEE models, adding adherence covariates did not change the interpretation of the reduction and rebound. Importantly, follow‐up exercise adherence was associated with a significantly lower T2D risk rebound (*β* = −0.08 per 10% adherence, *p =* 0.024), whereas adherence main effects were not statistically significant for MACE models. Results from time‐window–specific models incorporating adherence covariates for both MACE and T2D risks are presented in Table 4.

**TABLE 4:** MACE and T2D risk models with adherence covariates (time‐window–specific analyses).

Model (adherence added)	Reduction (Post‐WL vs. Baseline) (coef, *p*)	Rebound (1 year vs. Post‐WL) (coef, *p*)	Adherence main effect (coef, *p*)	Time × Group (*p*)	Adherence × Group (*p*)
MACE					
WL exercise adherence	−0.14, < 0.001	—	−0.05, 0.218	0.081	0.023
Follow‐up exercise adherence	—	+0.16, < 0.001	−0.01, 0.634	0.485	0.447
Follow‐up diet adherence	—	+0.16, < 0.001	−0.01, 0.742	0.307	0.633
T2D					
WL exercise adherence	−0.51, 0.025	—	+0.00, 0.945	0.735	0.492
Follow‐up exercise adherence	—	+0.84, 0.024	−0.08, 0.024	0.158	0.196
Follow‐up diet adherence	—	+0.89, 0.109	−0.09, 0.173	0.136	0.452

*Note:* Coefficients are *β* on the logit (log‐odds) scale from fractional logit GEE. Adherence is modeled per 10 percentage point increase. Weight loss (WL) exercise adherence was modeled using Baseline and Post‐WL only; follow‐up exercise adherence and follow‐up diet adherence were modeled using Post‐WL and 1 year only.

## 4 Discussion

In this cohort of healthy premenopausal women with overweight, we observed that weight loss consistently resulted in substantial improvement in predicted 10‐year risk for both T2D and MACE across all three intervention arms. However, T2D and MACE risk scores rebounded during the year after weight loss concomitant with weight regain, such that the net changes from Baseline were diminished compared with the immediate Post–WL improvements. This overall pattern is consistent with the well‐described tendency for cardiometabolic benefits of lifestyle‐ and obesity medication‐induced weight loss to diminish when weight is partially regained, even when individuals remain below their baseline weight [34, 35].

When considering MACE risk specifically, the reduction from Baseline to Post‐WL was significant for all groups, though the magnitude of this reduction was smaller than that observed for T2D, and the rebound from Post‐WL to 1‐year follow‐up was again significant for all groups, resulting in no significant change compared with Baseline. It is noteworthy that this cohort consisted of relatively young, generally healthy premenopausal women, who have relatively low baseline CMDS risk for MACE. Additionally, because MACE risk is calculated based upon contemporaneous BMI, BP, lipids, and glucose, the predicted probability naturally reflects the combined contribution of adiposity‐related and metabolic risks associated with insulin resistance. As a result, risk reduction may track closely with the weight loss phase, and when weight and risk factors partially rebound, the composite estimate will also tend to drift back toward baseline.

Although the overall MACE trend was similar across groups, Diet‐only achieved a larger Baseline‐to–Post‐WL reduction than Diet+Resistance. This is consistent with the idea that MACE risk is only minimally affected by resistance exercise. Alternatively, the ability of resistance training during energy restriction to preserve lean mass and influence BMI, hemodynamics, and lipid metabolism may be beneficial in ways that are not fully captured by the CMDS multivariable risk model [36, 37]. For example, exercise can improve cardiovascular health indirectly through factors such as endothelial function, fitness level, or autonomic tone, which are not accounted for by standard clinical risk predictors [38]. Similarly, the absence of significant race differences in MACE risk change was driven by effects of the interventions on the major modifiable factors (i.e., BMI, BP, lipid, and glucose levels) which trended in a similar direction throughout weight reduction and partial weight regain. At the same time, a null between‐race difference in MACE risk change does not preclude meaningful race‐related differences in individual biomarkers [39].

The effects of these interventions on T2D risk were somewhat different than observed for MACE. All groups experienced larger absolute reductions in the 10‐year risk of T2D after weight loss, indicating that weight loss has a greater effect to diminish T2D risk than MACE as captured by risk factors incorporated into validated CMDS equations. During follow‐up, the largest amount of rebound occurred within the Diet‐only group, while increments in risk at 1 year in Diet+Aerobic and Diet+Resistance were less marked, and Diet+Resistance was the only group to maintain a significant net improvement. Large lifestyle trials show that achieving and maintaining weight loss improve diabetes‐related risk factors, but that partial or full weight regain is generally associated with deterioration of glycemic control and other cardiometabolic traits [7, 35, 40]. In this context, the significant sustained improvement at 1 year in the Diet+Resistance group could reflect the role of this type of exercise in preserving or increasing skeletal muscle mass and strength, which may be valuable in supporting glucose disposal capacity and metabolic resilience during weight regain, even if the group‐level differences in risk rebound were not significant after correction [36, 37].

Exercise modality might contribute to differences in diabetes‐related risk trajectories through different mechanisms. Aerobic training typically produces strong improvements in cardiorespiratory fitness and insulin sensitivity, while resistance training enhances or maintains lean mass and sometimes improves glucose metabolism through increases in muscle quantity and quality. Randomized trials in individuals with established T2D show that both aerobic and resistance training improve glycemic control, with combined training showing the greatest reduction in HbA1c levels [17, 41]. Prospective studies also suggest that there is a link between weight training and a reduced incidence of T2D, with combined aerobic and resistance exercise having the most beneficial effect [36, 42]. However, to accurately compare exercise modalities, energy balance should be taken into consideration. Since many trials do not tightly equilibrate body weight across training modalities, it might be difficult to attribute differences in glycemic outcomes to the type of exercise rather than to differences in weight trajectories.

Race‐stratified results for T2D risk showed significant Baseline‐to–Post‐WL improvement for both EA and AA. However, baseline risk for T2D was higher in AA but reached the same nadir as in EA as a result of the interventions. Then, following the 1‐year period of weight regain, T2D risk rose in AA but not in EA. These within‐race patterns may indicate different rebound dynamics between AA and EA, possibly reflecting variations in weight regain, fat distribution, changes in fitness, and unmeasured contextual factors influencing long‐term behavior and physiology.

Our adherence‐adjusted analyses further support the interpretation that maintaining intervention behaviors during follow‐up matters for sustaining metabolic improvement. In particular, higher follow‐up exercise adherence was associated with lower T2D risk rebound, while follow‐up exercise and diet adherence were not significant in MACE models. This pattern is biologically plausible because glycemic regulation is highly responsive to changes in skeletal muscle glucose uptake, mitochondrial function, and fitness, all of which are maintained by continued exercise exposure. These findings highlight that continued exercise following weight reduction may be particularly important for preventing metabolic risk rebound, even when some weight regain occurs.

Strengths of this study include the randomized assignment to diet‐only versus diet plus aerobic or resistance training, the use of the standardized low‐calorie diet during the WL phase in a controlled feeding format, and the highly structured supervision of exercise training. Furthermore, the longitudinal design included repeated measures at Baseline, Post‐WL, and at 1 year, along with the standardization of inpatient measurements in the follicular phase. Another strength is the application of CMDS, which combines multiple cardiometabolic risk factors into single quantitative scores validated to predict T2D and MACE. Additionally, subjects in this study underwent a 4‐week weight stabilization prior to each evaluation visit, during which meals were provided to maintain weight stability. This reduces the impact of acute metabolic effects that might result from overfeeding or caloric restriction, which might influence glycemic and lipid profiles, independent of underlying cardiometabolic risk. The use of fractional logit models fit with GEE appropriately accommodates repeated measures, so that group‐ or race‐specific trajectories can be estimated while controlling for within‐participant correlation. Finally, the time‐window–specific adherence models provide additional insight into whether individual behavior variability helps explain changes in predicted risk.

However, this study also has some limitations. First, this analysis defines risk estimates rather than the hard outcomes of overt diabetes and cardiovascular events. Second, the participants in this study were healthy and sedentary premenopausal women with overweight, which will limit the generalizability of our results to men, older individuals, people with obesity, and those with established cardiometabolic disease. Additional studies are needed to replicate these findings in individuals with obesity (BMI ≥ 30 kg m^−2^), as the amount of risk reduction and the modality‐specific effects of exercise may differ. Third, because not all participants provided data at every time point, our population‐average GEE estimates assume that missing follow‐up data were MAR. However, if participants were lost to follow‐up because of unobserved health changes or their underlying risk trajectory, then the estimated risk changes could be biased. Future work should investigate the robustness of findings to missing data using sensitivity analysis techniques such as inverse probability weighting or pattern mixture models. Fourth, follow‐up exercise participation was encouraged but not required, producing adherence heterogeneity that can weaken intention‐to‐treat differences between exercise arms, particularly at 1 year. Fifth, variations in weight change trajectories which were minimized but did occur may make it more difficult to identify the weight‐independent benefits of individual interventions. Finally, the predicted risks in this study were calculated using equations developed and validated in large prospective cohorts. Despite the fact that CMDS has been validated in both AA and EA, risk prediction models may not perform accurately when applied to populations different from the ones they were developed in.

In this cohort of healthy premenopausal women with overweight, diet‐induced weight loss with or without aerobic or resistance exercise resulted in significant improvements in 10‐year risk of MACE and T2D. However, these risks rose over the 1‐year follow‐up period accompanied by weight regain. The one exception is that lower risk scores for T2D were sustained in those randomized to Diet+Resistance despite the weight regain. These data support the importance of maintaining structured exercise and weight loss following diet and exercise interventions and indicate that resistance exercise may have particular benefits in weight loss interventions aiming to prevent progression to T2D.

## Author Contributions

A.M.E. analyzed the data and drafted the manuscript; G.R.H. oversaw data collection and contributed to study design and manuscript drafting; C.M. contributed to study design and manuscript drafting; G.F. contributed to study design and manuscript drafting; W.T.G. validated the use of the CMDS score and contributed to study design and manuscript drafting; C.R.H. validated the use of the CMDS score and contributed to study design and manuscript drafting. All authors revised and approved the final manuscript.

## Funding

This investigation was supported by the following grants from the National Institutes of Health: R01 DK51684, R01 DK 49779, M01‐RR00032, and P30‐DK56336.

## Conflicts of Interest

The authors declare no conflicts of interest.

## Supporting information


**Figure S1:** CONSORT flow diagram showing participant flow in the baseline‐risk analytic sample across Baseline, Post‐WL, and 1 year, with group‐specific counts at each interval. Dropout indicates participants who withdrew or were lost to follow‐up before the subsequent assessment. Missing indicates participants who attended the visit but were missing one or more variables required to estimate predicted 10‐year MACE/T2D risk. Enter indicates participants with follow‐up risk observed but missing baseline risk (excluded from baseline‐risk–based analyses). Baseline risk missing but follow‐up observed: Post‐WL only *n* = 1, Post‐WL + 1 year *n* = 8.
**Table S1:** Baseline predicted risk and study completeness.
**Figure S2:** Up‐Set plot showing participant data completeness across study time points. Bar plots show the number of participants with available predicted 10‐year MACE/T2D risk at Baseline, Post‐WL, and 1 year in the main cohort (*n* = 212) and among entrants (*n* = 9). The distribution of completeness patterns is shown, including entrants who contributed data starting at Post‐WL (Post‐WL only; Post‐WL + 1 year).

## Data Availability

The data that support the findings of this study are available on request from the corresponding author. The data are not publicly available due to privacy or ethical restrictions.
