# Supplementary material for: Weight Loss Improves Predicted 10‐Year MACE and Type 2 Diabetes Risk, but Benefits Partially Rebound at 1 Year
Source: Obesity (Silver Spring). 2026 Aug 23;34(10):1869–79. doi: 10.1002/oby.70291 (PMC13615421; doi:10.1002/oby.70291)
Supplement: Supplementary file 1 [file OBY-34-1869-s001.docx]

**Figure S1.** CONSORT flow diagram showing participant flow in the baseline-risk analytic sample across Baseline, Post-WL, and 1-year, with group-specific counts at each interval. Dropout indicates participants who withdrew or were lost to follow-up before the subsequent assessment. Missing indicates participants who attended the visit but were missing one or more variables required to estimate predicted 10-year MACE/T2D risk. Enter indicates participants with follow-up risk observed but missing baseline risk (excluded from baseline-risk–based analyses). Baseline risk missing but follow-up observed: Post-WL only n=1, Post-WL+1-year n=8.

**Table S1. Baseline predicted risk and study completeness.**

| Analysis (baseline-risk–based) | Baseline only, (n=85) | Baseline+Post-WL, (n=30) | Baseline+Post-WL+1y, (n=92) | Overall P (3 groups) | Pairwise P values (2 vs 1; 3 vs 1; 3 vs 2) | Dropout before Post-WL, P (pseudo-R²) | Dropout before 1-year, P (pseudo-R²) |
| --- | --- | --- | --- | --- | --- | --- | --- |
| **MACE risk** | 0.80 (0.72-0.89) | 0.87 (0.71-1.03) | 0.84 (0.76-0.92) | 0.800 | 0.470; 0.555; 0.730 | 0.492 (0.0016) | 0.713 (0.0010) |
| **T2D risk** | 13.0 (11.9-14.2) | 12.3 (10.3-14.4) | 12.8 (11.8-13.9) | 0.736 | 0.556; 0.797; 0.668 | 0.575 (0.0011) | 0.650 (0.0015) |

Baseline predicted MACE and T2D risks expressed as % (95% CI) shown by completeness group. Overall p-values test for differences across the three groups; pairwise p-values compare groups using post-estimation marginal contrasts. Logistic regression models assessed whether baseline predicted risk was associated with dropout before Post-WL or before 1-year follow-up; model fit is summarized using McFadden pseudo-R².

 Dropout before 1-year models were estimated among participants with baseline+Post-WL data available. *Baseline predicted risk was not associated with completeness (no differences in baseline risk across completeness groups) and did not predict dropout before Post-WL or before 1-year follow-up (all p-values non-significant; pseudo-R² ≈ 0).*

**Figure S2.** Up-Set plot showing participant data completeness across study timepoints. Bar plots show the number of participants with available **predicted 10-year MACE/T2D risk** at Baseline, Post-WL, and at 1-year in the main cohort (n=212) and among entrants (n=9). The distribution of completeness patterns is shown, including entrants who contributed data starting at Post-WL (Post-WL only; Post-WL+1-year).
